# Just try it: A six months protocol for borderline personality disorder impulsivity and symptoms reduction

**DOI:** 10.1192/j.eurpsy.2021.1182

**Published:** 2021-08-13

**Authors:** S. Martin

**Affiliations:** Clinical Psychology, Sylvia MARTIN- Psycho.tcce, - NIMES, France

**Keywords:** Impulsivity, cognitive behavioral therapy, Borderline personality disorder

## Abstract

**Introduction:**

Borderline Personality Disorder is defined from its impulsivity issues regarding relationships, abandonment and rejection issues and emotional regulation problems. This personality disorder issue are hard to treat and often related to poor treatment outcomes. Nonetheless, Dialectical Behavioral Therapy stands as a great therapeutical approach that can be adapted.

**Objectives:**

We tested a 6-month CBT protocol (ECCCLORE)-3 modules respectively working on emotion regulation, distress tolerance and relationships- in a French context to compare its effectiveness to treatment as Usual (TAU).

**Methods:**

We recruited 56 patients suffering from BPD, 34 receiving ECCCLORE treatment and 22 receiving TAU. We assessed BPD traits, impulsivity with UPPS, aggressiveness with AQ12, Suicidal risk with SBQr and Hopelessness with H.

**Results:**

Our results revealed the effectiveness of this 6 months DBT adaptation for decreasing BPD traits and most of clinical dimensions. The dynamic analysis revealed the mediating effect of AQ12.

**Conclusions:**

Shortened treatment protocol are effective for reducing symptoms. Further research is needed to replicate these results.

